# Children's Cortisol and Cell-Free DNA Trajectories in Relation to Sedentary Behavior and Physical Activity in School: A Pilot Study

**DOI:** 10.3389/fpubh.2019.00026

**Published:** 2019-02-27

**Authors:** Christoph Becker, Sebastian Schmidt, Elmo W. I. Neuberger, Peter Kirsch, Perikles Simon, Ulrich Dettweiler

**Affiliations:** ^1^Department of Sport and Health Sciences, Technical University of Munich, Munich, Germany; ^2^Faculty of Social Science, Media and Sport, Johannes Gutenberg University, Mainz, Germany; ^3^Department of Clinical Psychology, Central Institute of Mental Health, Medical Faculty Mannheim, University of Heidelberg, Heidelberg, Germany; ^4^Department of Cultural Studies and Languages, Faculty of Arts and Education, University of Stavanger, Stavanger, Norway

**Keywords:** cortisol, cfDNA, physical activity, health, outdoor environment, Bayesian inference

## Abstract

The worldwide prevalence of mental disorders in children and adolescents increased constantly. Additionally, the recommended amount of physical activity (PA) is not achieved by this age group. These circumstances are associated with negative impacts on their health status in later life and can lead to public health issues. The exposure to natural green environments (NGE) seems to be beneficial for human health. The compulsory school system offers great opportunities to reach every child with suitable health-related contents and interventions at an early stage. The concept of Education Outside the Classroom (EOtC) uses NGE and sets focus on PA. Therefore, EOtC might be a beneficial educational intervention to promote students health. The association between biological stress markers and sedentary behavior (SB) plus PA is insufficiently evaluated in school settings. This exploratory study aims to evaluate the association between students' cortisol, plus circulating cell-free deoxyribonucleic acid (cfDNA) levels, and their SB, light PA (LPA), and moderate-to-vigorous PA (MVPA). We assessed data from an EOtC program (intervention group [IG], *n* = 37; control group [CG], *n* = 11) in three seasons (fall/spring/summer) in outdoor lessons (IG) in a NGE and normal indoor lessons (CG). SB and PA were evaluated by accelerometry, and cortisol and cfDNA levels by saliva samples. Fitted Bayesian hierarchical linear models evaluated the association between cortisol and cfDNA, and compositional SB/LPA/MVPA. A steady decline of cortisol in the IG is associated with relatively high levels of LPA (posterior mean = −0.728; credible interval [CRI 95%]: −1.268; −0.190). SB and MVPA tended to exhibit a similar effect in the CG. A high amount of cfDNA is positively associated with a relatively high amount of SB in the IG (posterior mean, 1.285; CRI: 0.390; 2.191), the same association is likely for LPA and MVPA in both groups. To conclude, LPA seems to support a healthy cortisol decrease in children during outdoor lessons in NGEs. Associations between cfDNA and SB/PA need to be evaluated in further research. This study facilitates the formulation of straightforward and directed hypotheses for further research with a focus on the potential health promotion of EOtC.

## Introduction

The upsurge in the worldwide prevalence of overweight and obesity in children is anticipated to reach 9.1% in 2020 (1), a high proportion of children do not reach the recommended levels of physical activity (PA) (2, 3), and suffer from mental disorders (4). Chronic stressful events could exert adverse impacts on brain development and result in major mental health-related problems in later life (5, 6). These circumstances require a need for action to improve children and adolescents health perspectives. Successful interventions should therefore consider that (1) PA and exercise during childhood is associated with the development of active lifestyles in later life, improved cognitive functions (7, 8) and, thus, with positive effects on health and prevention of common diseases (9), (2) the exposure to natural green environments (NGE) can have beneficial health effects [see e.g., (10, 11)], and that (3) children spend a substantial share of their waking hours in school. Therefore, the compulsory school system in western countries offers excellent opportunities to reach every child and adolescent with specific interventions focusing on PA in NGE to improve children and adolescents health perspectives.

The present paper aims to address these topics by investigating the relation between biological stress responses and physical activity in students taught in two different school settings: an indoor setting and an outdoor setting in a NGE. Here, we extend our original investigation (12) by increasing our set of dependent variables and introducing circulating cell-free deoxyribonucleic acid (cfDNA) as an innovative biological marker, sedentary behavior (SB) and light physical activity (LPA) as more differentiated measures of physical activity and by applying advanced statistical models to better describe relations between our measures.

Both cortisol and cfDNA are important biomarkers in relation to stress, SB and PA. Thus, the comparison of both cortisol and cfDNA in relation to students' relative levels of SB and PA is a promising approach to investigate students' biological stress response in different school settings. Important findings on cortisol and cfDNA in relation to physical and psychosocial stressful situations are therefore outlined.

Recent studies have focused on exploring the construct of “stress” and its potential negative association with health (13). In fact, an individual's physiological and psychological response, assessed by different stress biomarkers or questionnaire items, could be correlated with several positively, as well as negatively, connoted stimuli. Koolhaas et al. (13) argued that the term “stress” should be restricted to situations of uncontrollability or unpredictability of stimuli which however, must be restricted to “psychological stress” and is not true for so called “physical stress” (14, 15), which can be defined as a loss of homeostasis induced by physical not psychological conditions. Examples of such uncontrollable situations in school are examinations, testimonials, increased mental loads or prolonged social pressure (16, 17). Such stressors can lead to an interruption of the regular circadian cortisol rhythm. The relevance of a normal diurnal cortisol rhythm with high levels of cortisol in the morning and a steady decline until evening has been widely investigated (18–20). Furthermore, several external stimuli could be involved in the disturbance of a normal diurnal cortisol rhythm, for instance, light pollution during nighttime, or continuous changes in waking hour schedules. Moreover, a dysfunctionality in the hypothalamic pituitary adrenocortical (HPA) axis as one primary biological stress system plays a crucial role. In a recent systematic literature review and meta-analysis, Adam et al. (21) reported that a chronic abnormal flat diurnal cortisol rhythm correlated with poor mental and physical health symptoms for various populations. Other experimental studies (22, 23) evaluated the association between cortisol levels and PA, with a particular focus on different PA intensities, as well as the diurnal cortisol rhythm. These studies revealed that high PA intensities ranging from 60 to 80% of the maximal oxygen uptake (VO2 max) (22) or 80% VO2 max (23) for, at least, 30 min resulted in statistically significant higher cortisol levels compared with resting control situations. Interestingly, participants' cortisol levels decreased, although not statistically significant, not only in the resting control groups (CG) but also during low PA intensities of 40% VO2 max. These studies illustrate the potential impact of PA on cortisol levels.

Besides the well-established but also critically discussed stress marker cortisol (24), the circulating cfDNA has garnered more importance as a potential physiological stress marker. Different mechanisms can result in the release of the cfDNA into the human plasma. While an increase in cfDNA levels because of classic cell-death mechanisms would take several hours, or even days, other more rapid mechanisms are related to exercise. Based on plasma samples, the cfDNA is a well-established indicator of the activation of the innate immunity. Various studies have revealed that the innate immunity could be activated by both psychologically (25, 26) and physiologically (27–32) stressful situations. In particular, the cfDNA has been proven to be highly sensitive to physical exercise as a stressor [see (27) for review]. Reportedly, the cfDNA increased with moderate PA below the level of the aerobic–anaerobic transition (29, 33). A recent study (30) reported that cortisol and plasma cfDNA levels positively correlated and both increased in participants under physiological and psychosocial stressful situations. Regarding psychological stress little is known about the reactivity of cfDNA concentrations. To date, only one study has reported that lowering psychological stress in women treated for infertility reduces the plasma cfDNA concentration, a notion that is principally in line with the concept of a stress-associated, sensitive proinflamatory marker (34). Furthermore, current research suggests that major depressive symptoms are associated with elevated levels of cfDNA (35, 36). Cianga et al. (37) studied cfDNA in saliva of immunosuppressed patients. The results indicate that the most important source of DNA in saliva samples are leukocytes that travel from the blood to the oral cavity, where they play an important role in protection against pathogens. The specific cells and tissues, which are involved in psychological induced cfDNA elevation, are still unknown. However, the effect of stress hormones on leukocyte profiles is well-documented in biomedical studies of mammals. This includes glucocorticoid-induced alterations in cell trafficking, or redistribution from blood to other body compartments [reviewed in Davis et al. (38)]. This furthermore indicates an indirect link between cortisol and cfDNA. Higher cortisol values were associated with a greater number of neutrophils (38). In response to infection, tissue injury or exercise, neutrophil glucocorticoids can produce extracellular traps, which are likely to contribute to the pool of cfDNA (39). Most research on the cfDNA is restricted to plasma samples and controlled laboratory settings. However, a study has reported that the cfDNA in the saliva and serum possess a similar half-life time and both follow a first-order clearance model (40). Furthermore, in both body fluids, the cfDNA seems to be predominantly released by cells of the hematopoietic lineage (31, 37). To the best of our knowledge, no research has investigated the association between cfDNA levels based on salivary samples and exercise in an experimental setting, at least, in schools.

SB and PA are relevant factors with different effects in relation to health (3, 41) and in addition potential confounders for cortisol and cfDNA. Therefore, recent research developments with respect to SB, PA and health are outlined. With regard to public health, the relevance of SB and LPA has gained more attention recently. The authors of a recent review (42) suggested that high values in sedentary time correlated with an increased risk of cardio-metabolic disease, decreased fitness, self-esteem, academic achievement, and pro-social behavior for children and adolescent. Very obviously there is also a relation between SB and mental health, particularly depression (43). LPA seems to be beneficial to reduce obesity, overall mortality risk and should be considered for inclusion in PA recommendations (44). Therefore, it is of great importance to consider all parts of human behavior and especially to account for the compositional nature of SB, LPA and moderate-to-vigorous physical activity moderate-to-vigorous PA (MVPA). This approach has been proposed in the recent years (45, 46).

A great responsibility for children's PA and health could be assigned to educational institutions and their schedules. Apparently, students' time in school and its environment play a crucial role. Typically, NGE seem to be beneficial for promoting children's PA (47, 48), mental well-being (47) and cognition (49, 50). The amount of time children being exposed to NGA seems to be important for various health outcomes. Therefore, questions arise how the exposure to NGA and being physically active in NGA can contribute to enhance students PA and stress response during school time.

In a recent systematic literature review (51), we assessed the effects of regular compulsory school and curriculum-based education outside the classroom [EOtC, (52)] programs, focusing on students' health, PA, social, and learning dimensions. EOtC often takes place in both NGE and cultural settings. Students seem to benefit regarding learning and social dimensions. However, only one study reported improved mental health status of boys (53) and two studies (54, 55) reported higher PA levels during days with EOtC compared with regular school days. Unfortunately, the methodological quality of the 13 included studies was mostly moderate or low. Moreover, a recent large-scale study (56) on EOtC reported that the MVPA levels were significantly higher during EOtC compared with regular school days. However, the codependency among students SB, LPA, and MVPA levels remained unclear in this study. Overall, the existing knowledge on effects of EOtC with regard to PA and health is limited, despite the mentioned potentials of this type of teaching setting. Especially in the Scandinavian countries the EOtC approach is widely spread, creating good opportunities for further research (57).

In our recent publication (12), we compared the cortisol levels of students taught by applying an outdoor curriculum in the forest with children taught in the standard school setting. We were primarily interested in assessing the effect of outdoor teaching on children's normal diurnal cortisol rhythms. We reported that students in the intervention group (IG) exhibited a steady decline of cortisol levels during EOtC, whereas no such effect was observed in students in the CG during regular school days; in fact, the effect was independent of students MVPA levels. However, we could not entirely elucidate the differences in students' cortisol levels. We believe that the partial secondary exploitation of the data presented in this study is justified by the new knowledge gained, as we analyzed the cortisol and cfDNA values concerning the compositional nature of SB and PA.

This exploratory, longitudinal analysis aims to evaluate the association between students' cortisol and cfDNA levels and their SB, LPA, and MVPA in outdoor and indoor classroom environments. Based on our previous research, we assumed that different relations exist between the CG and the IG with respect to their cortisol response and PA. Specifically, we hypothesized that a decrease in students' cortisol levels can be explained by their compositional levels in SB, LPA and MVPA and explored if similar relationships exist for students cfDNA response.

## Materials and Methods

### Study Design and Intervention

This exploratory analysis is part of the research project “1 year in the forest–the influence of regular outdoor lessons in a natural environment on biological indicators of stress resilience.” The research in the NGE comprised a great complexity concerning measurement procedures and confounding factors. Thus, in this project, we applied a mixed-methods approach in a prospective, longitudinal quasi-experimental design. In addition, functional magnetic resonance imaging, saliva cortisol and saliva cfDNA, three-axis accelerometry, and constructs of the Self-Determination Theory were used as described by Dettweiler et al. (12).

This intervention study was conducted at a secondary school in Heidelberg, Germany. Since the school year 2013–2014, a group of fifth-grade students were taught one compulsory school day per week for the entire schoolyear in a nearby forest. The pedagogical concept of the forest teaching setting was inspired by the Scandinavian udeskole/uteskole approach as well as outdoor education from New Zealand [see (57–59) for further details]. Thus, teachers intended to facilitate student-centered, hands-on, and experimental learning situations in close connection to the NGE. In addition, this change of space within the physical setting of the “classroom” implied different opportunities for problem-solving, co-operation, experimentation, and to be physically active on students' free choice during the lessons. Furthermore, students undertook regular walks to reach specific places in the forest. Of note, the contents of the lessons in the forest setting were highly connected to the formal school curriculum and were taught in cross-disciplinary units on the forest days, including a certain variance concerning the practical relevance and season. Moreover, subject-by-subject teaching was applied on standard school days for both the IG and the CG based on traditional indoor teaching concepts [refer Dettweiler et al. (12) for further information regarding timetables and Von Au (60) for the pedagogical concept].

### Participants and Data Collection

We enrolled participants from fifth and sixth grades from the school year 2014–2015. In this school year, three fifth-grade classes had forest teaching, and only one fifth-grade class had regular indoor teaching. Owing to this administrative decision of the school, we could not enroll the same number of fifth-grade students in the IG and CG. Thus, we enrolled students from a sixth-grade regular indoor teaching class into the CG; these students did not participate in the forest teaching setting during their fifth-grade school year 2013–2014. Overall, we enrolled 48 students in this study (IG, 37; CG, 11). As some students were absent during the school year, we could not collect datasets from all 48 students at all-time points in fall, spring, and summer. Furthermore, not all saliva samples provided adequate material for analysis, and accidentally acceleration sensors got lost. Of note, descriptive and enrollment data for participants is presented elsewhere (12).

We collected both samples for saliva cfDNA and cortisol using Salivette™/Cortisol- Salivette™ collection tubes (Sarstedt, Nümbrecht, Germany) at time points 08:30 a.m., 10:30 a.m., and 12:30 p.m. during the seasons fall, spring and summer. All participants were told not to eat 15 min prior every data collection. Saliva cfDNA levels were evaluated using undiluted saliva according to the protocol described elsewhere (61). After centrifuging at 1,600 × g for 2 min (room temperature), the supernatant was transferred into a new collection tube and frozen at −20°C before measurement. In addition, salivary samples for cortisol quantification were frozen at −20°C immediately after the arrival at the Biopsychology Laboratory, Technical University Dresden, and cortisol levels were determined using a commercially available luminescence immunoassay (IBL, Hamburg, Germany). Based on the validation study by Khoury et al. (62), we applied the summary indices peak reactivity (PR) and the area under the curve with respect to increase (AUCi). (For further details regarding the calculation and application of the summary indices, refer to Fekedulegn et al. (63), Khoury et al. (62), and Pruessner et al. (64) and the Supplementary Material, section Material and Methods).

We determined both SB and PA of the IG and CG using triaxial Axivity AX3 acceleration sensors (Axivity Ltd., Newcastle upon Tyne, UK). One sensor was attached to each child's back above the upper point of the posterior iliac crest, with the aid of a medical tape (56, 65). The sensors were worn between 08:30 a.m. and 12:30 p.m. during school time. All children were instructed not to re-attach the sensor to their skin once it fell off. All sensors were initialized at 100 Hz and ±8G bandwidth. In addition, we converted the raw vector magnitude acceleration data to ActiLife file format by an in-house software developed by the University of Southern Denmark. Children's PA levels were analyzed using ActiLife v.6.11.4 (ActiGraph, Pensacola, FL). In addition, cut-off points reported by Romanzini et al. (66) were used to distinguish SB, LPA, and MVPA; these cut-off points have been proven to exhibit a good validity among children and adolescents to identify patterns of SB, LPA, and MVPA. However, the validity and comparability of acceleration sensors, as well as applied cut-off points, have been controversially discussed. Therefore, certain differences have to be considered when comparing studies on SB and PA, especially effects of varying epoch lengths, wear time algorithms, and activity cut-points (67–69).

### Statistical Analyses

In studies on PA and health, one specific behavior is often analyzed independently from other behaviors. Recent studies focused on this issue and reported that human behavior during a finite time of the day needs to be recognized as a composition that accumulates to 100% of that time. Thus, the components (e.g., sleep, SB, LPA, MVPA) are perfectly codependent and an approach that considers all parts of the composition is recommended to provide reliable evidence on human behaviors related to health (45, 46).

To set up, document and run the Bayesian hierarchical linear models (BHLMs), to evaluate associations between students' cortisol and cfDNA levels, respectively, and their relative time spent in SB, LPA, and MVPA, we applied the software packages ggthemes (70), jagsUI (71), rjags (72), and R2jags (73) in R 3.4.1 (2017-06-30) (74). The usual way to fit regression models with compositional covariates is to apply isometric log-ratio (ilr) or centered log-ratio (clr) transformations on raw values, which is justified as the parts of a composition perfectly correlate and standard regression techniques result in multicollinearity problems. However, the use of ilr or clr transformations poses problems with the interpretation, as the meaning of posterior parameter values remains unclear, especially in hierarchical models. Thus, in the given analysis, a Bayesian ridge regression version suggested by Parnell (75), which accepts raw compositions, was implemented and the raw composition values were transformed into a matrix using a common prior distribution function.

The likelihood for the applied BHLMs reads

$$\begin{array}{l} Y_{i}\sim N(\alpha_{i{\text{d}}_{i}}+\beta_{{\text{cmp}}_{i}}\left(grp_{i}\times x_{\left[1:3\right]i}\right)\\ +\beta_{ssn_{i}}x_{4_{i}}+\beta_{gdr_{i}}x_{5_{i}}+\beta_{t_{i}}x_{6_{i}},\sigma_{y}^{2}),\;for\;i=\;1,\;\ldots n \end{array}$$

where

$$\begin{array}{l} x_{\left[1:3\right]i}=\left(\begin{array}{ccc} SB_{1} & LPA_{1} & MVPA_{1}\\ SB_{2} & LPA_{2} & MVPA_{2}\\ \ldots & \ldots & \ldots \\ \ldots & \ldots & \ldots \\ SB_{n} & LPA_{n} & MVPA_{n} \end{array}\right) \end{array}$$

denotes the matrix of the composition of the three activity behaviors, and Table 1 presents the prior distributions of parameters in the cortisol and cfDNA models, respectively.

**Table 1:** The prior distribution of parameters for Bayesian hierarchical linear models.

**Cortisol (BHLM 1 and 3)**	**cfDNA (BHLM 2 and 4)**
${\alpha_{id}}_{j}\sim N\left(0,\sigma_{\alpha }^{2}\right)$	${\alpha_{id}}_{j}\sim N\left(0,\sigma_{\alpha }^{2}\right)$
_β_*cmp*_*j*_~*N*(μ_α_, 1)	_β_*cmp*_*j*_~*N*(μ_α_, 1)
μ_α_~ *N*(0, 5)	$\mu_{\alpha }\sim \;N\left(0,\;1^{-6}\right)$
_β_*t*_*j*_~*N*(0, 5)	${\beta_{t}}_{j}\sim N\left(0,\;1^{-6}\right)$
_β_*gdr*_*j*_~*N*(0, 5)	${\beta_{gdr}}_{j}\sim N\left(0,\;1^{-6}\right)$
_β_*ssn*_*j*_~*N*(0, 5)	${\beta_{ssn}}_{j}\sim N\left(0,\;1^{-6}\right)$
$\sigma_{y}^{2}\sim \;H∁\left(0,\;5\right)$	$\sigma_{y}^{2}\sim H∁\left(0,\;25\right)$
$\sigma_{\alpha }^{2}\sim H∁\left(0,\;5\right)$	$\sigma_{\alpha }^{2}\sim H∁\left(0,\;25\right)$

*id, identification of participants; cmp, composition; t, time point (midmorning, noon); gdr, gender (female; male); ssn, season (fall; spring; summer)*.

Furthermore, we applied a different set of priors for the respective cortisol and cfDNA models, which is justified to (a) address the well-established high-variance cortisol displays (within subjects over the course of the day with higher variance later in the day, within subjects at different seasons, and between subjects and gender) and (b) as to the best of our knowledge nothing is known about children's cfDNA levels in the saliva with respect to the daytime, season, gender, SB, and PA. In this study, we allowed random intercepts (α) for each id, and put a hyper prior to α centered to zero (i.e., inform the prior from the data). In addition, we centered βcmp on μα to tie the slope parameter βcmp to the random intercepts (equivalent to nesting ids in the groups); this is called “alternative hierarchical centering” and is an elegant way to borrow strength (i.e., statistical power) from an individual intercept and group. Putting this prior information on the composition dissolves the problem of collinearity, which is typically addressed in ilr- or clr-transformations, however without changing the scale of the output. Thus, the estimates could be interpreted straightforwardly. Finally, other priors were set to be normally distributed parameters around zero, with vaguely informed standard deviation for cortisol and super-vague informed standard deviation for cfDNA. Hence, the cfDNA model should be considered as a strictly provisional “reference model” (76).

In our analysis, we used log-transformed cortisol and cfDNA measures because of skewness and kurtosis (cf. Supplementary Table 1 and Supplementary Figures 3–8). Furthermore, the Markov chains were set to 50,000 iterations, a burn-in phase of 25,000, and a thinning-rate of 10 were applied.

## Results

We fitted different BHLMs to assess the possible impact of the relative amounts of SB, LPA, and MVPA on students' cortisol and cfDNA levels. The BHLMs for cortisol and cfDNA differed between the applied indices PR and AUCi. In the BHLM 2 and 4 (AUCi values), the covariates group (CG/IG), gender (female/male) and season (fall/spring/summer) were included. In the BHLM 1 and 3 (PR values), the covariate time point (midmorning/noon) was additionally included. In addition, we evaluated the model fit by means of the deviance information criterion (DIC). The convergence of the Markov chains were investigated by posterior predictive checks (cf. Supplementary Figures 7–10 for details; only the results for the respective best fitting BHLMs are presented).

Supplementary Figures 1–6 show the descriptive statistics for the variables cortisol and cfDNA, separated for the overall mean values, PR and AUCi and split by season and group. A stronger decrease of the cortisol values from 08:30 a.m. to 12:30 p.m. can be observed in IG compared to the CG, especially in the season's spring and summer. The cfDNA values show different patterns across the seasons and between the groups, which does not allow for a clear tendency. The values in fall and summer are higher compared to spring in both groups. Furthermore, only in the summer season the values of the CG are clearly higher compared to the IG. No clear correlations were found for cortisol and cfDNA with respect to group and season (cf. Supplementary Figure 11).

Supplementary Table 2 shows the descriptive statistics for the variables SB, LPA, and MVPA, separated for seasons and groups. We observed no evident differences between the arithmetic mean and the compositional mean in this study. Most apparent differences were observed in higher relative means of SB for the CG compared to the IG and lower relative means of MVPA for the CG compared to the IG. We neither observed any evident differences in seasons and the relative means of LPA.

### Association Between Cortisol PR/AUCi and SB/PA

**Figure 1:**
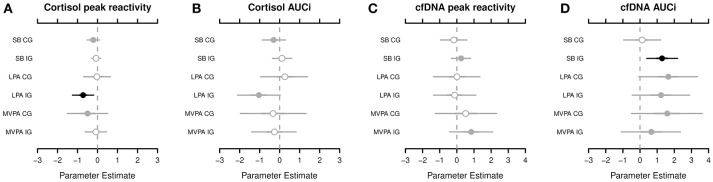
The highest probability density for associations among the cortisol peak reactivity **(A)**, cortisol area under the curve with respect to increase (AUCi) **(B)**, circulating cell-free deoxyribonucleic acid (cfDNA) peak reactivity **(C)**, and cfDNA AUCi **(D)**, respectively, and sedentary behavior (SB), light physical activity (LPA), and moderate-to-vigorous physical activity (MVPA), segregated by groups (CG, control group; IG, intervention group). Shading, whether 50% of credible interval (CRI; gray with open circle), 95% CRI (gray with closed circle), or neither (black) do overlap 0. Black dots indicate of a strong association between the dependent and indepentend variable. For example, cortisol peak reactivity and LPA in the IG—the adverse effect of the relative amounts of LPA on the cortisol peak reactivity applies to, at least, 95% of children in the posterior distribution.

According to the Markov chain Monte Carlo (MCMC) posterior distributions (cf. Figure 1 and Table 2 for summary and Supplementary Tables 3–4 for details), we observed a strong negative association in the IG for the relative amounts of LPA on the cortisol PR levels (posterior mean = −0.728; lower 95% credible interval [CRI]: −1.268; upper CRI: −0.190). In the CG, tendencies of a negative association were noted between SB and MVPA in the cortisol PR. Regarding cortisol AUCi, we observed the likelihood of a negative association in the IG for LPA and for a negative association in the CG for SB. Considering both posterior mean values and CIs, the IG exhibited stronger associations compared to the CG.

**Table 2:** The MCMC output of posterior probabilities.

**Variable**	**Model l**	**PM**	**SD**	**CRI 2.5%**	**CRI 25%**	**CRI 50%**	**CRI 75%**	**CRI 97.5%**	**Rhat**	**ESS**
SB (CG)	BHLM 1: cortisol PR	−0.215	0.152	−0.512	–**0.318**	–**0.213**	–**0.112**	0.085	1.001	7,500
LPA (IG)	BHLM 1: cortisol PR	−0.728	0.271	–**1.268**	–**0.908**	–**0.726**	–**0.551**	–**0.190**	1.001	7,500
MVPA (CG)	BHLM 1: cortisol PR	−0.499	0.524	−1.517	–**0.860**	–**0.501**	–**0.141**	0.527	1.001	5,900
SB (CG)	BHLM 2: cortisol AUCi	−0.293	0.298	−0.880	–**0.494**	–**0.291**	–**0.095**	0.297	1.001	7,100
LPA (IG)	BHLM 2: cortisol AUCi	−1.027	0.550	−2.112	–**1.403**	–**1.030**	–**0.661**	0.062	1.001	6,700
SB (IG)	BHLM 3: cfDNA PR	0.242	0.289	−0.329	**0.049**	**0.246**	**0.435**	0.801	1.001	5,400
MVPA (IG)	BHLM 3: cfDNA PR	0.839	0.636	−0.418	**0.416**	**0.839**	**1.269**	2.088	1.001	7,500
SB (IG)	BHLM 4: cfDNA AUCi	1.285	0.464	**0.390**	**0.970**	**1.286**	**1.595**	**2.191**	1.001	7,500
LPA (CG)	BHLM 4: cfDNA AUCi	1.643	0.877	−0.072	**1.058**	**1.652**	**2.232**	3.348	1.001	4,300
LPA (IG)	BHLM 4: cfDNA AUCi	1.231	0.858	−0.455	**0.647**	**1.227**	**1.804**	2.899	1.001	5,300
MVPA (CG)	BHLM 4: cfDNA AUCi	1.574	1.053	−0.492	**0.853**	**1.588**	**2.294**	3.632	1.001	3,600
MVPA (IG)	BHLM 4: cfDNA AUCi	0.649	0.889	−1.102	**0.053**	**0.658**	**1.251**	2.356	1.001	7,500

*BHLMs 1–4: variables are presented if lower 25% CRI and upper 75% CRI do not overlap 0; the respective values that do not overlap zero are bold. MCMC, Markov chain Monte Carlo; BLHM, Bayesian hierarchical linear model; SB, sedentary behavior; LPA, light physical activity; MVPA, moderate-to-vigorous physical activity; CG, control group; cfDNA, circulating cell-free deoxyribonucleic acid; IG, intervention group; PR, peak reactivity; AUCi, area under the curve with respect to increase; PM, posterior mean; SD, standard deviation; CRI, credible interval; Rhat, potential scale reduction factor; ESS, effective sample size; the ESS of the posterior distribution differs in relation to the convergence of the MCMC algorithms; the ESS depends on how accurately the proposed model fits the data*.

### Association Between cfDNA PR/AUCi and SB/PA

Compared with the cortisol PR and AUCi, the results of the cfDNA PR and AUCi were different. In the IG, we observed the likelihood of a positive association between SB and MVPA in the cfDNA PR. In fact, a strong positive association was noted in the IG for SB (posterior mean, 1.285; lower CRI: 0.390; upper CRI: 2.191) and tendencies of a positive association of LPA and MVPA in the cfDNA AUCi. In the CG, tendencies of a positive association were found for LPA and MVPA in the cfDNA AUCi (cf. Figure 1 and Table 2 for summary and Supplementary Tables 5–6 for details).

## Discussion

### General Observations

We conducted the present study to provide an update on the associations between students' cortisol levels and their physical activity as reflected in the measures of SB, LPA and MVPA as well as associations between students' cfDNA levels and their SB, LPA, and MVPA in outdoor and indoor classroom environments. While interpreting the results of this study, one must consider the character of this exploratory study: the specific school setting in which both (a) the number of available participants is low because of the situation of EOtC in Germany, and (b) the number of possibly uncontrolled confounders is high because of the real-world scenario. However, we believe that our study can provide valuable insights into the EOtC research, health promotion in schools, and the assessment and analysis of cortisol, cfDNA, SB, and PA in the educational setting. In our previous study (12), we reported a statistically significant difference in the measured cortisol levels between the CG and IG; regular teaching in the forest correlated with a lower cortisol secretion at noon compared with the standard indoor teaching, and this association was independent of students' MVPA levels. Considering the compositional nature and, thus, the codependency of students' SB, LPA, and MVPA, we elucidated students' cortisol values during school time in this study. Furthermore, we compared those results with associations between students' cfDNA levels and their SB and PA.

According to the presented posterior distributions of the four BHLMs, the associations between students' cortisol/cfDNA levels and their compositional amount of SB, LPA, and MVPA are diverse. Furthermore, the presented effects with respect to posterior means and credible intervals must be considered as small. First, we could partially confirm our previously reported results (12) reflecting their independence of the analysis methodology, as students' cortisol levels were not affected by the relative amounts of MVPA in the IG. However, in the CG, the relative amount of MVPA is more likely to exert a lowering effect on the cortisol PR; the more active the students were in MVPA levels, the more their cortisol levels seemed to decrease. Two experimental studies (22, 23) reported that human behaviors similar to SB and LPA correlated with declining cortisol levels, which is in concordance with a typical healthy diurnal rhythm. The lowering effect of LPA on cortisol in the IG therefore corroborates Hill et al. (22) and VanBruggen et al. (23), although the specific PA intensities are not directly comparable. Thus, it could be argued that the so-called “green effect” (12, 77) in the forest (positive effects of the NGE on humans' psychological well-being) supports the lowering physiological effect of relatively high LPA levels of cortisol to some extent. Perhaps, this supportive effect could be missing during the regular indoor teaching because of the built environment. The association between SB and cortisol is more likely for the CG but not for the IG. Of note, uncomfortable sitting situations in the forest could result in psychological stress in terms of discomfort or inability to concentrate, and, therefore, potentially be attributed to this missing association in the IG. The validation study by Khoury et al. (62) reported that the PR and AUCi indices exhibit similar results regarding the cortisol increase/decrease. In this study, most associations of SB/LPA/MVPA in cortisol PR/cortisol AUCi, respectively, exhibited similar tendencies; only the tendency for a negative association of MVPA in the CG was not present for the AUCi index. Thus, we assume that our cortisol dataset based on the measurement procedure with three time points (08:30 a.m., 10:30 a.m., and 12:30 p.m.) is not entirely comparable with the time points used previously (62).

Some studies have reported that cfDNA levels already increase with moderate PA below the level of the aerobic–anaerobic transition (29, 33). The results of the cfDNA AUCi posterior distributions suggest that such an association is also more likely in both teaching settings. However, the relative amount of SB in the IG also exhibits a strong positive association with students' cfDNA values, which, perhaps, cannot be easily explained on a theoretical or empirical basis. Furthermore, the deviance values in both cfDNA PR/AUCi analyses are rather high compared with the respective cortisol values (cf. Supplementary Tables 3–6), which is an indication that the cfDNA MCMCs present a worse convergence compared with the cortisol MCMCs. Regarding the cfDNA, the log-likelihood is lower, and the data deviate more substantially from the models assumptions compared with cortisol. Thus, a strong positive association of SB in students' cfDNA could be likely attributed to an overestimation in the model. Regarding cfDNA results, similar tendencies have been observed between the applied PR and AUCi indices for SB and MVPA in the IG. Both indices, PR and AUCi, seem to be stable for cortisol, whereas the results for the cfDNA are more diverse regarding the PR and AUCi; this could be potentially explained by the factor “time point.” As one's cortisol secretion follows a time-dependent diurnal rhythm (with expected high values in the morning and a steady decline from the noon to the evening), both validated indices account for the variation of cortisol over the period of the school day. Regarding the cfDNA, the AUCi index seems to better account for the less time-dependent and more PA-related secretion, which could be illustrated with nearly two times as much deviance for the cfDNA PR compared with the AUCi (cf. Supplementary Tables 5–6). In general, our analysis with the applied PR and AUCi indices was optimized for cortisol with its time-dependent diurnal rhythm and is therefore more appropriate to be used for cortisol compared with cfDNA.

Owing to the underlying pedagogical concept in the forest (60), students might have more breaks between phases of SB, LPA, and MVPA. Recent research has reported about positive health effects of breaks during extended periods of SB (78–81). The possible relevance of the number of interruptions is an interesting phenomenon to be evaluated in future research concerning students' SB and PA in school. According to the pedagogical concept of the present intervention (60), it could be hypothesized that students in the forest have more freedom to choose whether they want to sit, walk, or run. During the normal indoor teaching, students frequently have to sit still for the entire 45 min in each lesson. Perhaps, the hypothesized freedom of choice could result in better physiological reactions within students' adaptive systems. These aspects warrant further investigations. In future large-scale, prospective studies, also aspects of the sunlight exposure (82, 83) and further possible confounders should be considered.

### Limitations and Future Directions

This pilot study has an exploratory character that is especially based on the relatively low number of available participants along with the mentioned high number of uncertainties in children's cortisol and cfDNA levels in relation to their SB and PA in indoor and outdoor teaching. Because of non-existing previous research in this field, a meaningful sample size calculation to determine desirable statistical power was not possible before data collection. The promising approach of retrospective design calculation (84) to inform the interpretation of gained results should be considered in general. However, this approach was not applicable in our study because of the lack of reliable external information regarding the real effect sizes. Moreover, “without relatively large sample sizes we are often precluded from saying anything precise about the size of the effect because the likelihood function is not very peaked in small samples” [(85), p. 63]. Therefore, with the results of this pilot study, gained with Bayesian inference, we do not aim to generalize our findings but rather inform prospective studies. In concordance with our previous analysis (12), three measurement points over 1 school-year provided only limited insight into the complex structure of regular compulsory outdoor lessons, students' levels of measured biological stress parameters, and the respective associations with their SB and PA. However, conducting more measurement days was not feasible for logistical and school organizational reasons. In addition, several acceleration sensors fell off because of warm weather conditions during the study time point summer, which resulted in the loss of PA data. Furthermore, we were not able to conduct a long-term cortisol analysis, as collected hair samples could not be analyzed [discussed in (12)]. Therefore, we were restricted to the measurements of saliva cortisol during the three time points at 8:30 a.m., 10:30 a.m., and 12:30 p.m. and need to assume that the salivary cortisol as an HPA axis biomarker reflects psychological and physiological induced stress with sufficient validity (24, 86). Furthermore, the allocation of students to the CG and IG was performed per the school policies and parents' choice. Thus, students could not be randomly allocated to a group by the experimenter, possibly implying certain bias. Hence, the overall small number of participants in this pilot study must be considered, and extensive, prospective studies are warranted to investigate further the tendencies explained in this study. In addition to cortisol and cfDNA the measurement of IGs should be considered in normal indoor settings. Furthermore, questionnaire-based assessment of students' mental well-being needs consideration.

## Conclusions

The most important finding is that despite little difference in LPA between the CG and the IG, relative long time spent in LPA in an outdoor teaching setting seems to be strongly associated with a decline of cortisol levels, whereas no such decrease can be observed in the indoor setting. This is of great importance for educational practice, as the combination of PA and the outdoor environment during EOtC seems to be beneficial for students stress response. Additionally, the observed relative amount of sedentary time is lower and that of MVPA higher in the outdoor teaching setting in a NGE compared with the indoor setting. That implies possible health benefits for students during EOtC. Additionally, a more clinically controlled study will elucidate children's cfDNA values in relation to SB and PA, as well as to cortisol. Furthermore, in future research concerning students' PA and health in school, the co-dependency of SB, LPA and MVPA should be taken into account. Future studies on EOtC can build on the gained knowledge to apply informed priors.

## Data Availability

The datasets for this study can be found in the Supplementary Material.

## Ethics Statement

This study was carried out in accordance with the recommendations of and was approved by the ethics committee of the Medical Faculty Mannheim, University of Heidelberg, Germany. The approval code is 2014-585N-MA. All subjects gave written informed consent in accordance with the Declaration of Helsinki.

## Author Contributions

PK, PS, and UD conceived and designed the study. CB collected the data. PS, SS, and EN developed tools to prepare the cfDNA-salvia probes which were statistically analyzed by CB and UD. CB wrote the paper with substantial contributions from all other authors. All authors proved the final version of the manuscript.

### Conflict of Interest Statement

The authors declare that the research was conducted in the absence of any commercial or financial relationships that could be construed as a potential conflict of interest.

## References

[1] de Onis M Blössner M Borghi E. Global prevalence and trends of overweight and obesity among preschool children. Am J Clin Nutr. (2010) 92:1257–64. 10.3945/ajcn.2010.29786 20861173

[2] Verloigne M Van Lippevelde W Maes L Yildirim M Chinapaw M Manios Y. Levels of physical activity and sedentary time among 10- to 12-year-old boys and girls across 5 European countries using accelerometers: an observational study within the ENERGY-project. Int J Behav Nutr Phys Activity (2012) 9:34. 10.1186/1479-5868-9-34 22462550 PMC3359200

[3] WHO Global Recommendations on Physical Activity for Health. Geneva: World Health Organisation (2010). 26180873

[4] Merikangas KR Nakamura EF Kessler RC. Epidemiology of mental disorders in children and adolescents. Dialogues Clin Neurosci. (2009) 11:7–20. 19432384 10.31887/DCNS.2009.11.1/krmerikangas PMC2807642

[5] Lupien SJ McEwen BS Gunnar MR Heim C. Effects of stress throughout the lifespan on the brain, behaviour and cognition. Nat Rev Neurosci. (2009) 10:434–45 10.1038/nrn2639 19401723

[6] Marin M-F Lord C Andrews J Juster R-P Sindi S Arsenault-Lapierre G. Chronic stress, cognitive functioning and mental health. Neurobiol Learn Memory (2011) 96:583–95. 10.1016/j.nlm.2011.02.016 21376129

[7] Erickson KI Hillman CH Kramer AF Physical activity, brain, and cognition. Curr Opin Behav Sci. (2015) 4:27–32. 10.1016/j.cobeha.2015.01.005

[8] Gomez-Pinilla F Hillman C. The influence of exercise on cognitive abilities. Compr Phys. (2013) 3:403–28. 10.1002/cphy.c110063 23720292 PMC3951958

[9] Janssen I LeBlanc AG. Systematic review of the health benefits of physical activity and fitness in school-aged children and youth. Int J Behav Nutr Phys Activity (2010) 7:40. 10.1186/1479-5868-7-40 20459784 PMC2885312

[10] Barton J Pretty J. What is the best dose of nature and green exercise for improving mental health? A multi-study analysis. Environ Sci Technol. (2010) 44:3947–55. 10.1021/es903183r 20337470

[11] Fong K Hart JE James P. A review of epidemiologic studies on greenness and health: updated literature through 2017. Curr Environ Health Rep. (2018) 5:77–87. 10.1007/s40572-018-0179-y 29392643 PMC5878143

[12] Dettweiler U Becker C Auestad BH Simon P Kirsch P. Stress in school. Some empirical hints on the circadian cortisol rhythm of children in outdoor and indoor classes. Int J Environ Res Public Health (2017) 14:475. 10.3390/ijerph14050475 28468292 PMC5451926

[13] Koolhaas JM Bartolomucci A Buwalda B de Boer SF Flügge G Korte SM. Stress revisited: a critical evaluation of the stress concept. Neurosci Biobehav Rev. (2011) 35:1291–301. 10.1016/j.neubiorev.2011.02.003 21316391

[14] Hackney AC. Stress and the neuroendocrine system: the role of exercise as a stressor and modifier of stress. Exp Rev Endocrinol Metabol. (2006) 1:783–92. 10.1586/17446651.1.6.783 20948580 PMC2953272

[15] McEwen BS Stress, definition and concepts of. In: Fink G. editor, Encyclopedia of Stress, 2nd ed, Vol. 1 Amsterdam: Academic Press p. 653 10.1016/B978-012373947-6.00364-0

[16] Raufelder D Kittler F Braun SR Lätsch A Wilkinson RP Hoferichter F The interplay of perceived stress, self-determination and school engagement in adolescence. School Psychol Int. (2014) 35:405–20. 10.1177/0143034313498953

[17] Torsheim T Aaroe LE Wold B. School-related stress, social support, and distress: prospective analysis of reciprocal and multilevel relationships. Scand J Psychol. (2003) 44:153–9. 10.1111/1467-9450.00333 12778983

[18] Gröschl M Rauh M Dörr H-G. Circadian rhythm of salivary cortisol, 17α-hydroxyprogesterone, and progesterone in healthy children. Clin Chem. (2003) 49:1688–91. 10.1373/49.10.1688 14500602

[19] Koch CE Leinweber B Drengberg BC Blaum C Oster H. Interaction between circadian rhythms and stress. Neurobiol Stress (2017) 6:57–67. 10.1016/j.ynstr.2016.09.001 28229109 PMC5314421

[20] Rotenberg S McGrath JJ Roy-Gagnon M-H Tu MT. Stability of the diurnal cortisol profile in children and adolescents. Psychoneuroendocrinology (2012) 37:1981–9. 10.1016/j.psyneuen.2012.04.014 22658393 PMC5760225

[21] Adam EK Quinn ME Tavernier R McQuillan MT Dahlke KA Gilbert KE. Diurnal cortisol slopes and mental and physical health outcomes: a systematic review and meta-analysis. Psychoneuroendocrinology (2017) 83:25–41. 10.1016/j.psyneuen.2017.05.018 28578301 PMC5568897

[22] Hill EE Zack E Battaglini C Viru M Viru A Hackney AC. Exercise and circulating cortisol levels: the intensity threshold effect. J Endocrinol Invest. (2008) 31:587–91. 10.1007/BF03345606 18787373

[23] VanBruggen MD Hackney AC McMurray RG Ondrak KS. The relationship between serum and salivary cortisol levels in response to different intensities of exercise. Int J Sports Physiol Perform. (2011) 6:396–407. 10.1123/ijspp.6.3.396 21911864

[24] Hellhammer DH Wüst S Kudielka BM. Salivary cortisol as a biomarker in stress research. Psychoneuroendocrinology (2009) 34:163–71. 10.1016/j.psyneuen.2008.10.026 19095358

[25] Marsland AL Walsh C Lockwood K John-Henderson NA. The effects of acute psychological stress on circulating and stimulated inflammatory markers: a systematic review and meta-analysis. Brain Behav Immunity (2017) 64:208–19. 10.1016/j.bbi.2017.01.011 28089638 PMC5553449

[26] Steptoe A Hamer M Chida Y. The effects of acute psychological stress on circulating inflammatory factors in humans: a review and meta-analysis. Brain Behav Immunity (2007) 21:901–12. 10.1016/j.bbi.2007.03.011 17475444

[27] Breitbach S Tug S Simon P. Circulating cell-free DNA. Sports Med. (2012) 42:565–86. 10.2165/11631380-000000000-00000 22694348

[28] Frühbeis C Helmig S Tug S Simon P Krämer-Albers E-M. Physical exercise induces rapid release of small extracellular vesicles into the circulation. J Extracell Vesicles (2015) 4:28239. 10.3402/jev.v4.28239 26142461 PMC4491306

[29] Haller N Tug S Breitbach S Jörgensen A Simon P. Increases in circulating cell-free DNA during aerobic running depend on intensity and duration. Int J Sports Physiol Perform. (2017) 12:455–62. 10.1123/ijspp.2015-0540 27617389

[30] Hummel EM Hessas E Müller S Beiter T Fisch M Eibl A. Cell-free DNA release under psychosocial and physical stress conditions. Transl Psychiatry (2018) 8:236. 10.1038/s41398-018-0264-x 30374018 PMC6206142

[31] Tug S Helmig S Deichmann E Schmeier-Jürchott A Wagner E Zimmermann T. Exercise-induced increases in cell free DNA in human plasma originate predominantly from cells of the haematopoietic lineage. Exer Immunol Rev. (2015) 21:164–73. 25826002

[32] Walsh NPG Shephard M Gleeson RJ Woods M Bishop JA Fleshner N Position statement. part one: immune function and exercise. Exer Immunol Rev. (2011) 17:6–63. 21446352

[33] Haller N Helmig S Taenny P Petry J Schmidt S Simon P. Circulating, cell-free DNA as a marker for exercise load in intermittent sports. PLoS ONE (2018) 13:e0191915. 10.1371/journal.pone.0191915 29370268 PMC5784997

[34] Czamanski-Cohen J Sarid O Cwikel J Levitas E Lunenfeld E Douvdevani A. Decrease in cell free DNA levels following participation in stress reduction techniques among women undergoing infertility treatment. Arch Women's Mental Health (2014) 17:251–3. 10.1007/s00737-013-0407-2 24420416

[35] Cai N Chang S Li Y Li Q Hu J Liang J. Molecular signatures of major depression. Curr Biol. (2015) 25:1146–56. 10.1016/j.cub.2015.03.008 25913401 PMC4425463

[36] Lindqvist D Fernström J Grudet C Ljunggren L Träskman-Bendz L Ohlsson L. Increased plasma levels of circulating cell-free mitochondrial DNA in suicide attempters: associations with HPA-axis hyperactivity. Transl Psychiatry (2016) 6:e971. 10.1038/tp.2016.236 27922635 PMC5315562

[37] Cianga Corina M Antohe I Zlei M Constantinescu D Cianga P Saliva leukocytes rather than saliva epithelial cells represent the main source of DNA. Revista Romana de Med de Laborator (2016) 24:31–44. 10.1515/rrlm-2016-0011

[38] Davis AK Maney DL Maerz JC The use of leukocyte profiles to measure stress in vertebrates: a review for ecologists. Func Ecol. (2008) 22:760–72. 10.1111/j.1365-2435.2008.01467.x

[39] Beiter T Fragasso A Hartl D Nieß AM. Neutrophil extracellular traps: a walk on the wild side of exercise immunology. Sports Med. (2015) 45:625–40. 10.1007/s40279-014-0296-1 25504501

[40] Yao W Mei C Nan X Hui L. Evaluation and comparison of in vitro degradation kinetics of DNA in serum, urine and saliva: a qualitative study. Gene (2016) 590:142–8. 10.1016/j.gene.2016.06.033 27317895

[41] Penedo FJ Dahn JR. Exercise and well-being: a review of mental and physical health benefits associated with physical activity. Curr Opin Psychiatry (2005) 18:189–93. 10.1097/00001504-200503000-00013 16639173

[42] Carson V Hunter S Kuzik N Gray CE Poitras VJ Chaput J-P. Systematic review of sedentary behaviour and health indicators in school-aged children and youth: an update. Appl Physiol Nutr Metabol. (2016) 41:240–65. 10.1139/apnm-2015-0630 27306432

[43] Stubbs B Vancampfort D Firth J Schuch FB Hallgren M Smith L. Relationship between sedentary behavior and depression: A mediation analysis of influential factors across the lifespan among 42,469 people in low- and middle-income countries. J Affect Disord. (2018) 229:231–8. 10.1016/j.jad.2017.12.104 29329054

[44] Füzéki E Engeroff T Banzer W. Health benefits of light-intensity physical activity: a systematic review of accelerometer data of the national health and nutrition examination survey (NHANES). Sports Med. (2017) 47:1769–93. 10.1007/s40279-017-0724-0 28393328

[45] Chastin SFM Palarea-Albaladejo J Dontje ML Skelton DA. Combined effects of time spent in physical activity, sedentary behaviors and sleep on obesity and cardio-metabolic health markers: a novel compositional data analysis approach. PLoS ONE (2015) 10:e0139984. 10.1371/journal.pone.0139984 26461112 PMC4604082

[46] Pedišić Ž Dumuid D Olds TS Integrating sleep, sedentary behaviour, and physical activity research in the emerging field of time-use epidemiology: definitions, concepts, statistical methods, theoretical framework, and future directions. Kinesiology (2017) 49:1–18. Available online at: https://hrcak.srce.hr/186506

[47] Kondo MC Fluehr JM McKeon T Branas CC. Urban green space and its impact on human health. Int J Environ Res Public Health (2018) 15:445. 10.3390/ijerph15030445 29510520 PMC5876990

[48] Tremblay M Gray C Babcock S Barnes J Bradstreet C Carr D. Position statement on active outdoor play. Int J Environ Res Public Health (2015) 12:6475. 10.3390/ijerph120606475 26062040 PMC4483712

[49] Bratman GN Hamilton JP Daily GC. The impacts of nature experience on human cognitive function and mental health. Ann N Y Acad Sci. (2012) 1249:118–36. 10.1111/j.1749-6632.2011.06400.x 22320203

[50] Dadvand P Nieuwenhuijsen MJ Esnaola M Forns J Basagaña X Alvarez-Pedrerol M. Green spaces and cognitive development in primary schoolchildren. Proc Natl Acad Sci USA. (2015) 112:7937–42. 10.1073/pnas.1503402112 26080420 PMC4491800

[51] Becker C Lauterbach G Spengler S Dettweiler U Mess F. Effects of regular classes in outdoor education settings. A systematic review on students' learning, social and health dimensions. Int J Environ Res Public Health (2017) 14:485. 10.3390/ijerph14050485 28475167 PMC5451936

[52] Nielsen G Mygind E Bølling M Otte CR Schneller MB Schipperijn J. A quasi-experimental cross-disciplinary evaluation of the impacts of education outside the classroom on pupils' physical activity, well-being and learning: the TEACHOUT study protocol. BMC Public Health (2016) 16:1117. 10.1186/s12889-016-3780-8 27776502 PMC5078947

[53] Gustafsson PE Szczepanski A Nelson N Gustafsson PA Effects of an outdoor education intervention on the mental health of schoolchildren. J Adv Educ Outdoor Learn. (2012) 12:63–79. 10.1080/14729679.2010.532994

[54] Mygind E A comparison between children's physical activity levels at school and learning in an outdoor environment. J Adv Educ Outdoor Learn. (2007) 2:161–76. 10.1080/14729670701717580

[55] Mygind E A comparison of childrens' statements about social relations and teaching in the classroom and in the outdoor environment. J Adv Educ Outdoor Learn. (2009) 9:151–69. 10.1080/14729670902860809

[56] Schneller MB Duncan S Schipperijn J Nielsen G Mygind E Bentsen P. Are children participating in a quasi-experimental education outside the classroom intervention more physically active? BMC Public Health (2017) 17:523. 10.1186/s12889-017-4430-5 28549469 PMC5446688

[57] Barfod K Ejbye-Ernst N Mygind L Bentsen P Increased provision of udeskole in Danish schools: an updated national population survey. Urban Forestry Urban Greening (2016) 20:277–81. 10.1016/j.ufug.2016.09.012

[58] Bentsen P Jensen FS Mygind E Randrup TB The extent and dissemination of udeskole in Danish schools. Urban Forestry Urban Greening (2010) 9:235–43. 10.1016/j.ufug.2010.02.001

[59] Von Au J Outdoor Education in Danish, Scottisch and German Schools - Competence Oriented and Context Specific Influences on Intentions and Actions of Experienced Outdoor Education Teachers [Outdoor Education an Schulen in Dänemark, Schottland und Deutschland – kompetenzorientierte und kontextspezifische Einflüsse auf Intentionen und Handlungen von Erfahrenen Outdoor Education-Lehrpersonen]. (Dr. phil Dissertation), Pädagogischen Hochschule Heidelberg, Heidelberg. (2017).

[60] Von Au J Outdoor Days - Learning with passion, hand and a clear mind [Draußentage - Lernen mit Herz, Hand und viel Verstand] Pädagogik (2018) 4:10–13.

[61] Breitbach S Tug S Helmig S Zahn D Kubiak T Michal M. Direct quantification of cell-free, circulating DNA from unpurified plasma. PLoS ONE (2014) 9:e87838. 10.1371/journal.pone.0087838 24595313 PMC3940427

[62] Khoury JE Gonzalez A Levitan RD Pruessner JC Chopra K Basile VS. Summary cortisol reactivity indicators: interrelations and meaning. Neurobiol Stress (2015) 2:34–43. 10.1016/j.ynstr.2015.04.002 26844238 PMC4721456

[63] Fekedulegn DB Andrew ME Burchfiel CM Violanti JM Hartley TA Charles LE. Area under the curve and other summary indicators of repeated waking cortisol measurements. Psychosomat Med. (2007) 69:651–9. 10.1097/PSY.0b013e31814c405c 17766693

[64] Pruessner JC Kirschbaum C Meinlschmid G Hellhammer DH. Two formulas for computation of the area under the curve represent measures of total hormone concentration versus time-dependent change. Psychoneuroendocrinology (2002) 28:916–31. 10.1016/S.0306-4530(02)00108-7 12892658

[65] Schneller MB Bentsen P Nielsen G Brønd JC Ried-Larsen M Mygind E. Measuring children's physical activity: compliance using skin-taped accelerometers. Med Sci Sports Exer. (2017) 49:1261–9. 10.1249/MSS.0000000000001222 28181981

[66] Romanzini M Petroski EL Ohara D Dourado AC Reichert FF. Calibration of actigraph GT3X, actical and RT3 accelerometers in adolescents. Eur J Sport Sci. (2014) 14:91–9. 10.1080/17461391.2012.732614 24533499

[67] Banda JA Haydel KF Davila T Desai M Bryson S Haskell WL. Effects of varying epoch lengths, wear time algorithms, and activity cut-points on estimates of child sedentary behavior and physical activity from accelerometer data. PLoS ONE (2016) 11:e0150534. 10.1371/journal.pone.0150534 26938240 PMC4777377

[68] Ried-Larsen M Brønd JC Brage S Hansen BH Grydeland M Andersen LB. Mechanical and free living comparisons of four generations of the Actigraph activity monitor. Int J Behav Nutr Phys Activity (2012) 9:113. 10.1186/1479-5868-9-113 22971175 PMC3463450

[69] Rowlands AV Fraysse F Catt M Stiles VH Stanley RM Eston RG. Comparability of measured acceleration from accelerometry-based activity monitors. Med Sci Sports Exer. (2015) 47:201–10. 10.1249/MSS.0000000000000394 24870577

[70] Arnold JB Ggthemes: Extra Themes, Scales and Geoms for ‘ggplot2’ (Version R package version 3.4.0). (2017). Available online at: https://CRAN.R-project.org/package=ggthemes

[71] Kellner K jagsUI: A Wrapper Around ‘rjags’ to Streamline ‘JAGS’ Analyses. (Version R package version 1.4.9) (2017). Available online at: https://CRAN.R-project.org/package=jagsUI. https://CRAN.R-project.org/package=R2jags

[72] Plummer M rjags: Bayesian Graphical Models using MCMC (Version R package version 4-6) (2016). Available online at: https://CRAN.R-project.org/package=rjags. https://CRAN.R-project.org/package=rjags

[73] Su YS Yajima M R2jags: Using R to Run ‘JAGS’ (Version R package version 0.5-7). (2015) Available online at: https://CRAN.R-project.org/package=R2jags. https://CRAN.R-project.org/package=R2jags

[74] R Development Core Team R: A Language and Environment for Statistical Computing (Version 3.4.1). Vienna, Austria: R Foundation for Statistical Computing. (2013). Available online at: http://www.R-project.org/

[75] Parnell A jags_compositional_covariates.R (2018). Available online at: https://github.com/andrewcparnell/jags_examples/blob/master/jags_scripts/jags_compositional_covariates

[76] Gelman A Hill J Data Analysis Using Regression and Multilevel/Hierarchical Models. Cambridge: Cambridge University Press (2006). 10.1017/CBO9780511790942

[77] Barton J Bragg R Wood C Pretty J Green Exercise Linking Nature, Health and Well-Being Vol. 1. London: Routledge (2016). 10.4324/9781315750941

[78] Chastin SFM Egerton T Leask C Stamatakis E. Meta-analysis of the relationship between breaks in sedentary behavior and cardiometabolic health. Obesity (2015) 23:1800–10. 10.1002/oby.21180 26308477

[79] Jalayondeja C Jalayondeja W Mekhora K Bhuanantanondh P Dusadi-Isariyavong A Upiriyasakul R. Break in sedentary behavior reduces the risk of noncommunicable diseases and cardiometabolic risk factors among workers in a petroleum company. Int J Environ Res Public Health (2017) 14:501. 10.3390/ijerph14050501 28486414 PMC5451952

[80] Mailey EL Rosenkranz SK Casey K Swank A. Comparing the effects of two different break strategies on occupational sedentary behavior in a real world setting: a randomized trial. Preven Med Rep. (2016) 4:423–8. 10.1016/j.pmedr.2016.08.010 27583200 PMC4995540

[81] Owen N Healy GN Matthews CE Dunstan DW. Too much sitting: the population-health science of sedentary behavior. Exer Sport Sci Rev. (2010) 38:105–13. 10.1097/JES.0b013e3181e373a2 20577058 PMC3404815

[82] Jung CM Khalsa SBS Scheer FAJL Cajochen C Lockley SW Czeisler CA. Acute effects of bright light exposure on cortisol levels. J Biol Rhythms (2010) 25:208–16. 10.1177/0748730410368413 20484692 PMC3686562

[83] Pagels P Wester U Söderström M Lindelöf B Boldemann C. Suberythemal sun exposures at swedish schools depend on sky views of the outdoor environments – possible implications for pupils' health. Photochem Photobiol. (2016) 92:201–7. 10.1111/php.12540 26480960

[84] Gelman A Carlin J. Beyond power calculations:assessing type S (Sign) and Type M (Magnitude) errors. Perspec Psychol Sci. (2014) 9:641–51. 10.1177/1745691614551642 26186114

[85] Etz A Introduction to the concept of likelihood and its applications. Adv Methods Pract Psychol Sci. (2018) 1:60–9. 10.1177/2515245917744314

[86] Keil MF. Salivary cortisol: a tool for biobehavioral research in children. J Pediatr Nursing (2012) 27:287–9. 10.1016/j.pedn.2012.02.003 22405849 PMC3335961

