# Supplementary material for: Children's Cortisol and Cell-Free DNA Trajectories in Relation to Sedentary Behavior and Physical Activity in School: A Pilot Study
Source: Front Public Health. 2019 Feb 27;7:26. doi: 10.3389/fpubh.2019.00026 (PMC6400867; doi:10.3389/fpubh.2019.00026)
Supplement: Supplementary file 1 [file Data_Sheet_1.docx]

Supplementary Material

Children’s Cortisol and Cell-Free DNA Trajectories in Relation to Sedentary Behavior and Physical Activity in School: A Pilot Study

**Christoph Becker*, Sebastian Schmidt, Elmo W.I. Neuberger, Peter Kirsch, Perikles Simon, Ulrich Dettweiler**

***Correspondence:** Christoph Becker: chris.becker@tum.de

# Supplementary Materials and Methods: Summary Indices Peak Reactivity and Area Under the Curve with Respect to Increase

# A wide range of summary indices is used in the literature for identifying the change in endocrinological parameters, especially, measuring the total production of cortisol (1, 2). Khoury et al. (3) evaluated 15 summary indices. These indices are often interrelated and the authors concluded that the abundance of 15 indices is unnecessary, and that the area under the curve with respect to ground (AUCg), the area under the curve with respect to increase (AUCi), and the peak reactivity (PR) are suitable indices for measuring multiple times per day. In order to account for cortisol/cell-free deoxyribonucleic acid (cfDNA) physiological complexity, we applied different indices for cortisol and cfDNA in the Bayesian hierarchical linear models (BHLM). Our indices take into account three cortisol/cfDNA measurement time points, 08:30 AM, 10:30 AM, and 12:30 PM, as well as the respective time interval of measured sedentary behavior (SB), light physical activity (LPA) and moderate-to-vigorous PA (MVPA). Similar to the calculations done by these authors (2, 3), we calculated PR using the difference between cortisol/cfDNA measurements at 10:30 AM compared with 8:30 AM (time point midmorning), and 12:30 PM compared with 10:30 AM (time point noon) and used this information for calculating SB/LPA/MVPA. AUCi was calculated according to the formulas presented by Pruessner et al. (1). The overall values of SB/LPA/MVPA for the time interval from 08:30 AM to 12.30 PM were used in the BHLMs for AUCi. AUCg was not calculated as we were primarily interested in the change over time and not in the overall secretion of cortisol/cfDNA.

# Supplementary Figures and Tables

## Supplementary Figures

**2.1.1 Boxplot of Overall log Cortisol Mean Values**


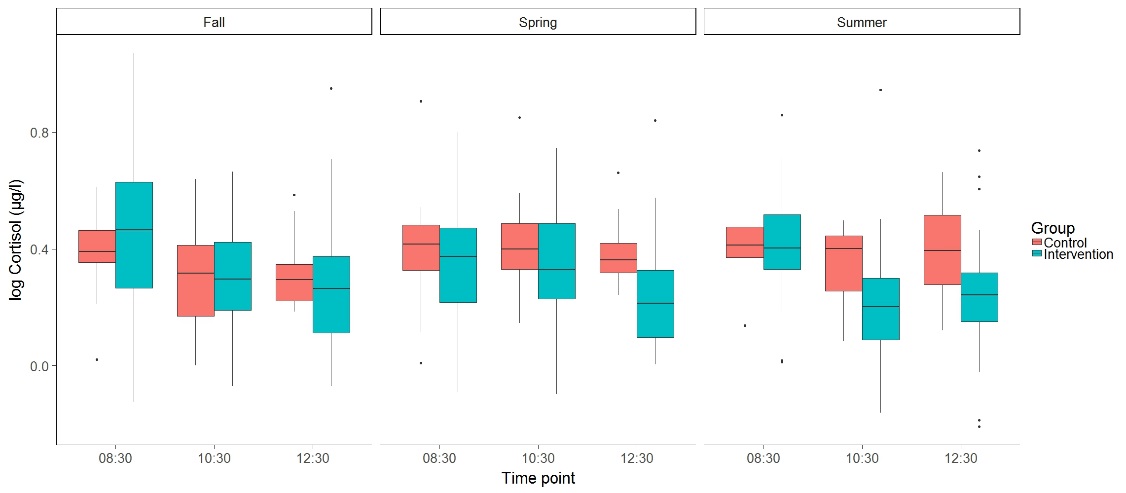


**Supplementary Figure 1.** Boxplot of log cortisol (µg/l) mean values, split by group (control, intervention), time point (08:30, 10:30, 12:30) and season (fall, spring, summer)

**2.1.2 Boxplot of log Cortisol PR Mean Values**


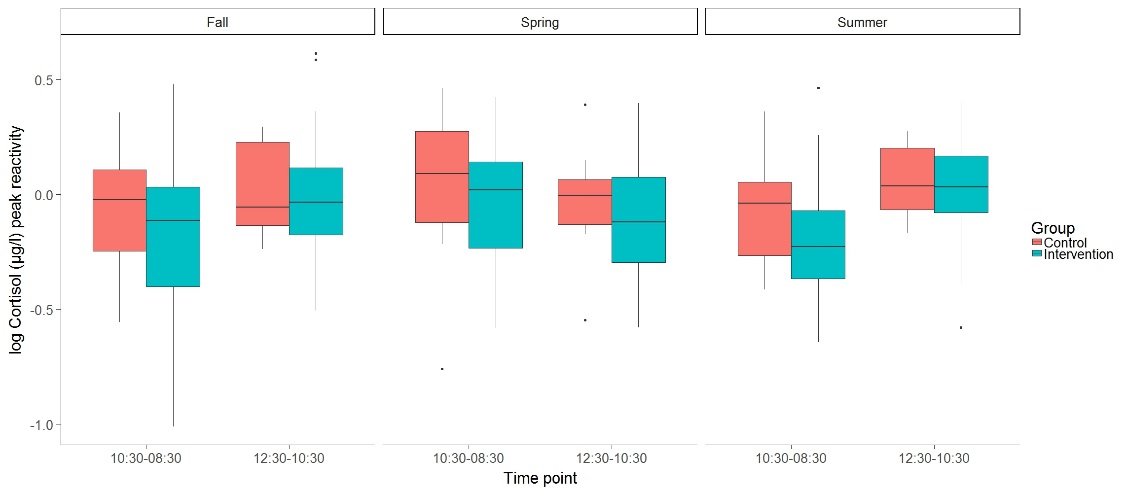


**Supplementary Figure 2.** Boxplot of log cortisol (µg/l) peak reactivity (PR) mean values, split by group (control, intervention), time point (10:30-08:30, 12:30-10:30) and season (fall, spring, summer)

**2.1.3 Boxplot of log Cortisol AUCi Mean Values**


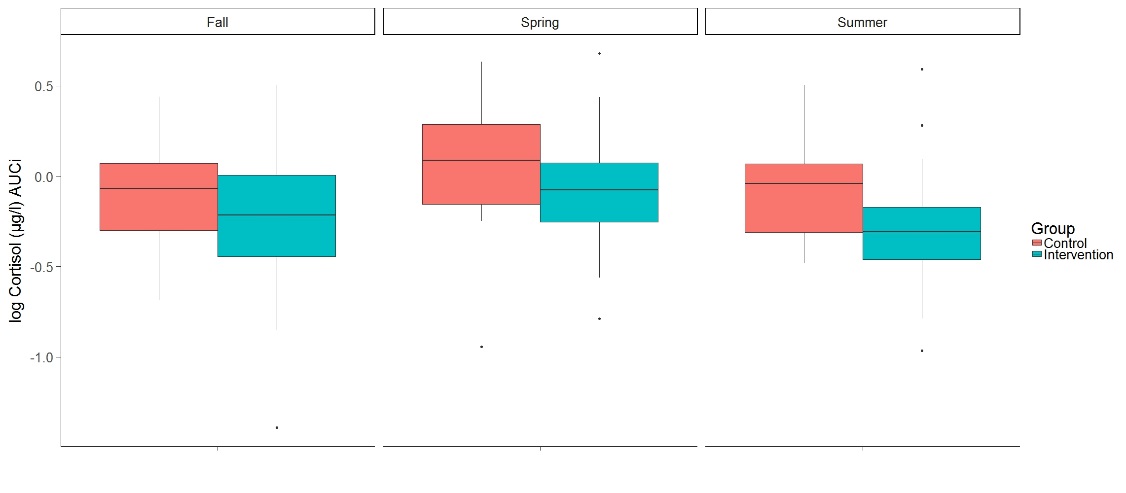


**Supplementary Figure 3.** Boxplot of log cortisol (µg/l) area under the curve with respect to increase (AUCi) mean values, split by group (control, intervention) and season (fall, spring, summer)

**2.1.4 Boxplot of overall log cfDNA Mean Values**


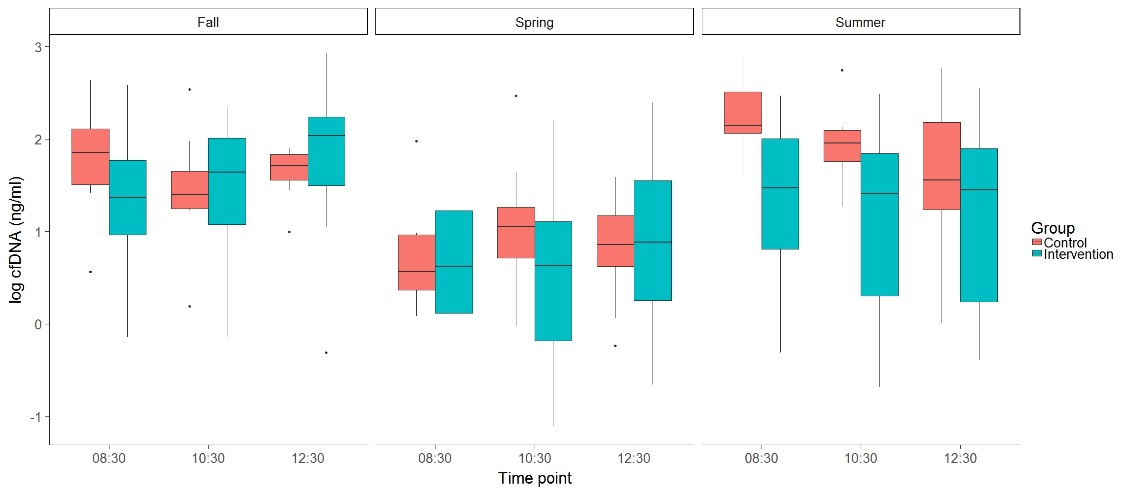


**Supplementary Figure 4.** Boxplot of log cfDNA (ng/ml) mean values, split by group (control, intervention), time point (08:30, 10:30, 12:30) and season (fall, spring, summer)

**2.1.5 Boxplot of log cfDNA PR Mean Values**
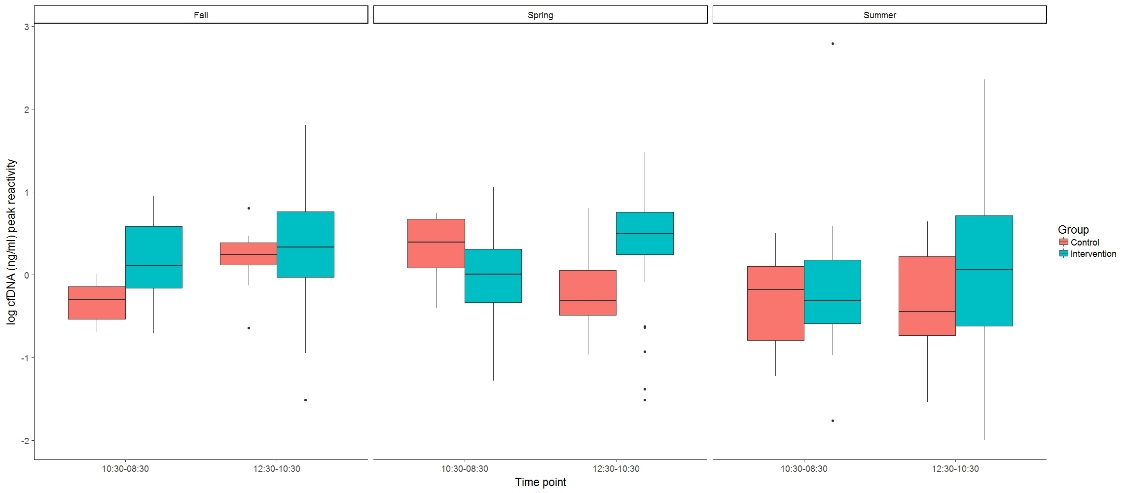


**Supplementary Figure 5.** Boxplot of log cfDNA (ng/ml) peak reactivity (PR) mean values, split by group (control, intervention), time point (10:30-08:30, 12:30-10:30) and season (fall, spring, summer)

**2.1.6 Boxplot of log cfDNA AUCi Mean Values**


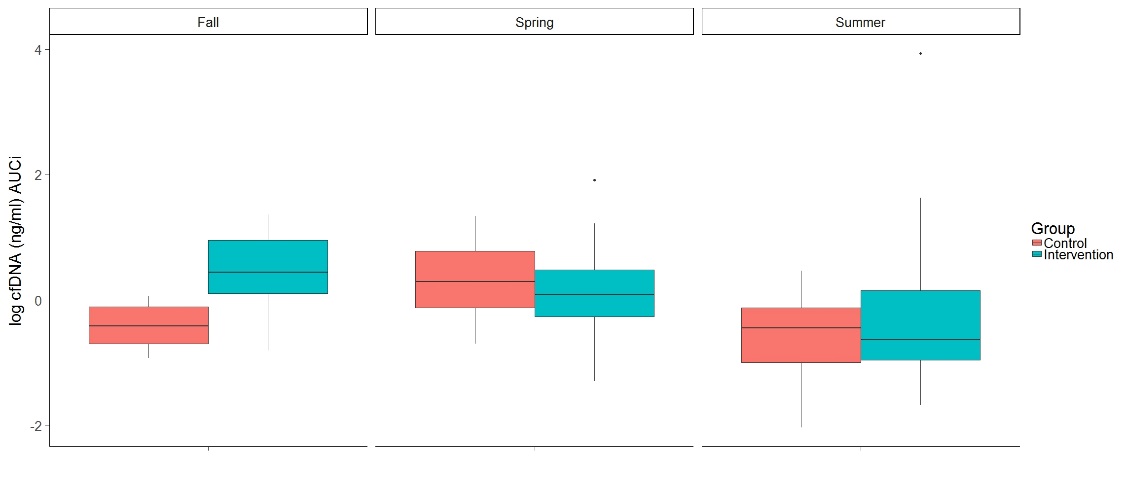


**Supplementary Figure 6.** Boxplot of log cfDNA (ng/ml) area under the curve with respect to increase (AUCi) mean values, split by group (control, intervention) and season (fall, spring, summer)

**2.1.7 Posterior Predictive Check for Bayesian Hierarchical Linear Model 1**

**Supplementary Figure 7.** Posterior predictive check for BHLM 1. Comparison of actual datasets and simulated datasets. Bayesian p-values close to 0.5 indicate that the simulated data is well distributed around the real observation.

**2.1.8 Posterior Predictive Check for Bayesian Hierarchical Linear Model 2**

**Supplementary Figure 8.** Posterior predictive check for BHLM 2. Comparison of actual datasets and simulated datasets. Bayesian p-values close to 0.5 indicate that the simulated data is well distributed around the real observation.

**2.1.9 Posterior Predictive Check for Bayesian Hierarchical Linear Model 3**

**Supplementary Figure 9.** Posterior predictive check for BHLM 3. Comparison of actual datasets and simulated datasets. Bayesian p-values close to 0.5 indicate that the simulated data is well distributed around the real observation.

**2.1.10 Posterior Predictive Check for Bayesian Hierarchical Linear Model 4**

**Supplementary Figure 10.** Posterior predictive check for BHLM 4. Comparison of actual datasets and simulated datasets. Bayesian p-values close to 0.5 indicate that the simulated data is well distributed around the real observation.

**2.1.11 Scatterplot log Cortisol – log cfDNA**


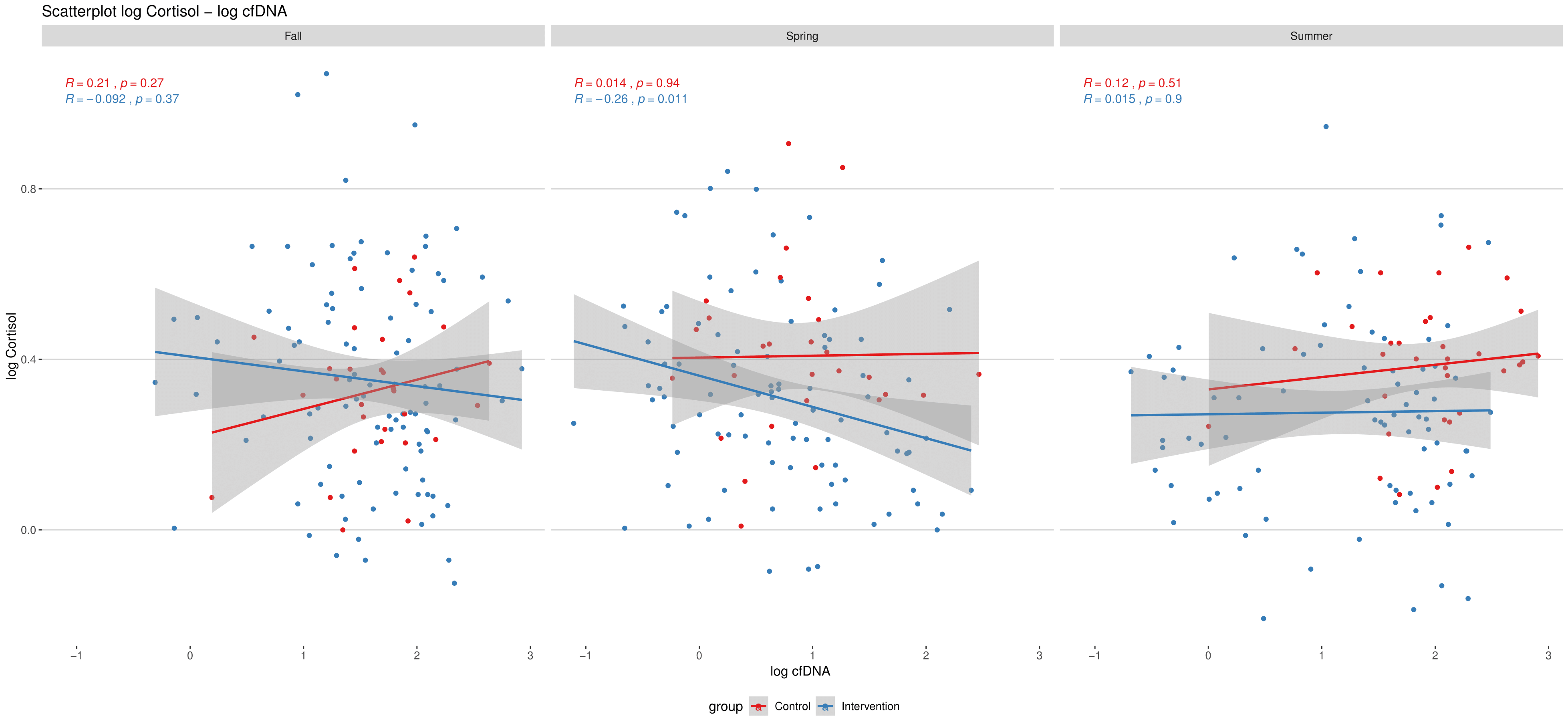


**Supplementary Figure 11**. Scatterplots of log Cortisol (µg/l) and log cfDNA (ng/ml) split by group (control, intervention) and season (fall, spring, summer)

## Supplementary Tables

**2.2.1 Descriptive Values for raw Cortisol, log Cortisol, sqrt Cortisol, raw cfDNA, log cfDNA and sqrt cfDNA**

|  | **raw Cortisol** | **log Cortisol** | **sqrt Cortisol** | **raw cfDNA** | **log cfDNA** | **sqrt cfDNA** |
| --- | --- | --- | --- | --- | --- | --- |
| **Mean** | 2.45 | 0.33 | 1.51 | 65.33 | 1.24 | 6.21 |
| **Std. Error of Mean** | 0.07 | 0.01 | 0.02 | 5.81 | 0.04 | 0.27 |
| **Std. Deviation** | 1.42 | 0.22 | 0.40 | 111.34 | 0.84 | 5.18 |
| **Variance** | 2.02 | 0.05 | 0.16 | 12397.16 | 0.71 | 26.82 |
| **Skewness** | 2.41 | 0.31 | 1.22 | 3.71 | -0.45 | 1.44 |
| **Kurtosis** | 9.29 | 0.28 | 2.67 | 17.38 | -0.62 | 2.67 |

**Supplementary Table 1.** Both log-transformed cortisol and cfDNA variables show the best values of distribution with regard to skewness and kurtosis in comparison to raw and square root transformed variables.

raw Cortisol = initial cortisol values; log Cortisol = log-transformed initial cortisol values; sqrt Cortisol = square root transformed initial cortisol values; cfDNA, circulating cell-free deoxyribonucleic acid; raw cfDNA = initial cfDNA values; log cfDNA = log-transformed initial cfDNA values; sqrt cfDNA = square root transformed initial cfDNA values

**2.2.2 Descriptive values for Sedentary Behavior, Light Physical Activity and Moderate-to-Vigorous Physical Activity**

|  | Fall CG | | | Spring CG | | | Summer CG | | |
| --- | --- | --- | --- | --- | --- | --- | --- | --- | --- |
|  | **SB** | **LPA** | **MVPA** | **SB** | **LPA** | **MVPA** | **SB** | **LPA** | **MVPA** |
| Arithmetic mean (SD) | 65.15 (7.28) | 27.29 (6.62) | 7.56 (2.90) | 57.80 (10.48) | 29.99 (5.14) | 12.21 (7.68) | 63.61 (12.02) | 25.66 (9.11) | 10.73 (4.32) |
| Compositional mean | 65.79 | 27.01 | 7.19 | 58.65 | 30.50 | 10.85 | 65.18 | 24.81 | 10.01 |
|  | **Fall IG** | | | **Spring IG** | | | **Summer IG** | | |
|  | **SB** | **LPA** | **MVPA** | **SB** | **LPA** | **MVPA** | **SB** | **LPA** | **MVPA** |
| Arithmetic mean (SD) | 50.19 (7.81) | 28.21 (4.95) | 21.59 (6.28) | 56.03 (8.86) | 25.68 (5.45) | 18.29 (6.51) | 51.78 (9.31) | 28.74 (5.09) | 19.48 (6.43) |
| Compositional mean | 50.58 | 28.34 | 21.07 | 56.75 | 25.74 | 17.51 | 52.17 | 28.97 | 18.86 |

**Supplementary Table 2.** Descriptive characteristics of arithmetic and compositional mean for SB, LPA, and MVPA. Results are presented in percentage of mean time in each behavior on the respective school day, segregated by seasons and groups.

SB, sedentary behavior; LPA, light physical activity; MVPA, moderate-to-vigorous physical activity; CG, control group; IG, intervention group; SD, standard deviation.

**2.2.2 MCMC Output for the BHLM 1: Cortisol PR**

|  | **PM** | **SD** | **CRI 2.5%** | **CRI 25%** | **CRI 50%** | **CRI 75%** | **CRI 97.5%** | **Rhat** | **n.eff** |
| --- | --- | --- | --- | --- | --- | --- | --- | --- | --- |
| SB (CG) | −0.215 | 0.152 | −0.512 | **−0.318** | **−0.213** | **−0.112** | 0.085 | 1.001 | 7500 |
| SB (IG) | –0.071 | 0.112 | –0.289 | –0.144 | –0.070 | 0.005 | 0.147 | 1.001 | 5600 |
| LPA (CG) | –0.032 | 0.339 | –0.699 | –0.257 | –0.032 | 0.193 | 0.635 | 1.001 | 7500 |
| LPA (IG) | –0.728 | 0.271 | **−1.268** | **−0.908** | **−0.726** | **−0.551** | **−0.190** | 1.001 | 7500 |
| MVPA (CG) | –0.499 | 0.524 | –1.517 | **−0.860** | **−0.501** | **−0.141** | 0.527 | 1.001 | 5900 |
| MVPA (IG) | –0.085 | 0.276 | –0.623 | –0.270 | –0.087 | 0.098 | 0.461 | 1.002 | 2200 |
| Season | 0.007 | 0.023 | –0.039 | –0.008 | 0.007 | 0.023 | 0.052 | 1.001 | 7500 |
| Gender male | 0.028 | 0.041 | –0.054 | 0.000 | 0.028 | 0.056 | 0.110 | 1.002 | 2100 |
| Time point noon | 0.079 | 0.041 | 0.000 | **0.052** | **0.079** | **0.107** | 0.160 | 1.001 | 7500 |
| Sigma | 0.265 | 0.013 | 0.241 | 0.256 | 0.264 | 0.273 | 0.291 | 1.001 | 6200 |
| Deviance | 39.402 | 4.902 | 31.117 | 35.975 | 38.908 | 42.325 | 50.507 | 1.001 | 7500 |

**Supplementary Table 3.** The MCMC output for the posterior probability for BHLM 1. DV = cortisol PR; IV = composition SB/LPA/MVPA; covariates group, season, gender, and time point. Values are bold if lower 25% CRI and upper 75% CRI do not overlap 0.

MCMC, Markov chain Monte Carlo; BLHM, Bayesian hierarchical-linear model; PR, peak reactivity; SB, sedentary behavior; LPA, light physical activity; MVPA, moderate-to-vigorous physical activity; CG, control group; IG, intervention group; season (fall, spring, summer); gender (female, male); time point (midmorning, noon); PM, posterior mean; SD, standard deviation; CRI, credible interval; Rhat, potential scale reduction factor; n.eff, effective sample size; DV, dependant variable; IV, indipendant variable

**2.2.3 MCMC Output for the BHLM 2: Cortisol AUCi**

|  | **PM** | **SD** | **CRI 2.5%** | **CRI 25%** | **CRI 50%** | **CRI 75%** | **CRI 97.5%** | **Rhat** | **n.eff** |
| --- | --- | --- | --- | --- | --- | --- | --- | --- | --- |
| SB (CG) | –0.293 | 0.298 | –0.880 | **−0.494** | **−0.291** | **−0.095** | 0.297 | 1.001 | 7100 |
| SB (IG) | 0.118 | 0.245 | –0.359 | –0.047 | 0.114 | 0.282 | 0.608 | 1.001 | 7500 |
| LPA (CG) | 0.246 | 0.596 | –0.919 | –0.156 | 0.250 | 0.643 | 1.404 | 1.001 | 7500 |
| LPA (IG) | –1.027 | 0.550 | –2.112 | **−1.403** | **−1.030** | **−0.661** | 0.062 | 1.001 | 6700 |
| MVPA (CG) | –0.316 | 0.826 | –1.947 | –0.880 | –0.316 | 0.235 | 1.321 | 1.002 | 1900 |
| MVPA (IG) | –0.258 | 0.581 | –1.400 | –0.649 | –0.267 | 0.136 | 0.895 | 1.002 | 2300 |
| Season | 0.014 | 0.037 | –0.057 | –0.011 | 0.014 | 0.038 | 0.085 | 1.001 | 4300 |
| Gender male | 0.046 | 0.098 | –0.147 | –0.020 | 0.047 | 0.111 | 0.239 | 1.001 | 6700 |
| Sigma | 0.288 | 0.027 | 0.241 | 0.270 | 0.286 | 0.305 | 0.347 | 1.002 | 2800 |
| Deviance | 37.777 | 14.434 | 12.005 | 27.371 | 36.864 | 47.052 | 69.210 | 1.001 | 3600 |

**Supplementary Table 4.** The MCMC output for the posterior probability for BHLM 2. DV = cortisol AUCi; IV = composition SB/LPA/MVPA; covariates group, season and gender. Values are bold if lower 25% CRI and upper 75% CRI do not overlap 0.

MCMC, Markov chain Monte Carlo; BLHM, Bayesian hierarchical-linear model; AUCi, area under the curve with respect to increase; SB, sedentary behavior; LPA, light physical activity; MVPA, moderate-to-vigorous physical activity; CG, control group; IG, intervention group; season (fall, spring, summer); gender (female, male); PM, posterior mean; SD, standard deviation; CRI, credible interval; Rhat, potential scale reduction factor; n.eff, effective sample size; DV, dependant variable; IV, indipendant variable

**2.2.4 MCMC Output for the BHLM 3: cfDNA PR**

|  | **PM** | **SD** | **CI 2.5%** | **CI 25%** | **CI 50%** | **CI 75%** | **CI 97.5%** | **Rhat** | **n.eff** |
| --- | --- | --- | --- | --- | --- | --- | --- | --- | --- |
| SB (CG) | –0.181 | 0.391 | –0.955 | –0.444 | –0.179 | 0.087 | 0.576 | 1.001 | 7500 |
| SB (IG) | 0.249 | 0.288 | –0.319 | **0.055** | **0.249** | **0.445** | 0.796 | 1.001 | 7500 |
| LPA (CG) | 0.009 | 0.689 | –1.356 | –0.448 | 0.007 | 0.480 | 1.346 | 1.001 | 4300 |
| LPA (IG) | –0.126 | 0.631 | –1.361 | –0.549 | –0.129 | 0.299 | 1.109 | 1.001 | 4800 |
| MVPA (CG) | 0.512 | 0.917 | –1.262 | –0.107 | 0.513 | 1.131 | 2.332 | 1.001 | 7500 |
| MVPA (IG) | 0.839 | 0.636 | –0.418 | **0.416** | **0.839** | **1.269** | 2.088 | 1.001 | 7500 |
| Season | –0.108 | 0.062 | –0.231 | **−0.150** | **−0.109** | **−0.066** | 0.015 | 1.001 | 6400 |
| Gender male | –0.147 | 0.110 | –0.362 | **−0.220** | **−0.147** | **−0.074** | 0.072 | 1.001 | 7500 |
| Time point noon | 0.253 | 0.108 | 0.038 | 0.181 | 0.254 | 0.327 | 0.461 | 1.001 | 7500 |
| Sigma | 0.675 | 0.035 | 0.611 | 0.651 | 0.674 | 0.698 | 0.748 | 1.001 | 7300 |
| Deviance | 411.222 | 4.392 | 404.037 | 408.108 | 410.696 | 413.810 | 421.139 | 1.001 | 7500 |

**Supplementary Table 5.** The MCMC output for the posterior probability for BHLM 3. DV = cfDNA PR; IV = composition SB/LPA/MVPA; covariates group, season, gender, and time point. Values are bold if lower 25% CRI and upper 75% CRI do not overlap 0.

MCMC, Markov chain Monte Carlo; BLHM, Bayesian hierarchical-linear model; cfDNA, circulating cell-free deoxyribonucleic acid; PR, peak reactivity; SB, sedentary behavior; LPA, light physical activity; MVPA, moderate-to-vigorous physical activity; CG, control group; IG, intervention group; season (fall, spring, summer); gender (female, male); time point (midmorning, noon); PM, posterior mean; SD, standard deviation; CRI, credible interval; Rhat, potential scale reduction factor; n.eff, effective sample size; DV, dependant variable; IV, indipendant variable

**2.2.4 MCMC Output for the BHLM 4: cfDNA AUCi**

|  | **PM** | **SD** | **CI 2.5%** | **CI 25%** | **CI 50%** | **CI 75%** | **CI 97.5%** | **Rhat** | **n.eff** |
| --- | --- | --- | --- | --- | --- | --- | --- | --- | --- |
| SB (CG) | 0.125 | 0.555 | –0.955 | –0.242 | 0.124 | 0.498 | 1.208 | 1.001 | 3800 |
| SB (IG) | 1.285 | 0.464 | **0.390** | **0.970** | **1.286** | **1.595** | **2.191** | 1.001 | 7500 |
| LPA (CG) | 1.643 | 0.877 | –0.072 | **1.058** | **1.652** | **2.232** | 3.348 | 1.001 | 4300 |
| LPA (IG) | 1.231 | 0.858 | –0.455 | **0.647** | **1.227** | **1.804** | 2.899 | 1.001 | 5300 |
| MVPA (CG) | 1.574 | 1.053 | –0.492 | **0.853** | **1.588** | **2.294** | 3.632 | 1.001 | 3600 |
| MVPA (IG) | 0.649 | 0.889 | –1.102 | **0.053** | **0.658** | **1.251** | 2.356 | 1.001 | 7500 |
| Season | –0.264 | 0.101 | **−0.461** | **−0.332** | **−0.262** | **−0.196** | **−0.067** | 1.001 | 7500 |
| Gender male | –0.283 | 0.186 | –0.651 | **−0.406** | **−0.285** | **−0.157** | 0.083 | 1.001 | 7500 |
| Sigma | 0.759 | 0.061 | 0.647 | 0.717 | 0.757 | 0.798 | 0.888 | 1.001 | 4100 |
| Deviance | 221.887 | 7.045 | 205.152 | 218.423 | 222.668 | 226.326 | 234.212 | 1.002 | 1200 |

**Supplementary Table 6:** The MCMC output for the posterior probability for BHLM 4. DV = cfDNA AUCi; IV = composition SB/LPA/MVPA; covariates group, season and gender. Values are bold if lower 25% CRI and upper 75% CRI do not overlap 0.

MCMC, Markov chain Monte Carlo; BLHM, Bayesian hierarchical-linear model; cfDNA, circulating cell-free deoxyribonucleic acid; AUCi, area under the curve with respect to increase; SB, sedentary behavior; LPA, light physical activity; MVPA, moderate-to-vigorous physical activity; CG, control group; IG, intervention group; season (fall, spring, summer); gender (female, male); PM, posterior mean; SD, standard deviation; CRI, credible interval; Rhat, potential scale reduction factor; n.eff, effective sample size; DV, dependant variable; IV, indipendant variable

References

1. Pruessner JC, Kirschbaum C, Meinlschmid G, Hellhammer DH. Two formulas for computation of the area under the curve represent measures of total hormone concentration versus time-dependent change. Psychoneuroendocrinology. 2002;28(7):916-931. doi: 10.1016/S0306-4530(02)00108-7

2. Fekedulegn DB, Andrew ME, Burchfiel CM, Violanti JM, Hartley TA, Charles LE, et al. Area Under the Curve and Other Summary Indicators of Repeated Waking Cortisol Measurements. Psychosomatic Medicine. 2007;69(7):651-659. doi: 10.1097/PSY.0b013e31814c405c

3. Khoury JE, Gonzalez A, Levitan RD, Pruessner JC, Chopra K, Basile VS, et al. Summary cortisol reactivity indicators: Interrelations and meaning. Neurobiology of Stress. 2015;2:34-43. doi: 10.1016/j.ynstr.2015.04.002
